# Supplementary material for: Lighten up the dark: metazoan parasites as indicators for the ecology of Antarctic crocodile icefish (Channichthyidae) from the north-west Antarctic Peninsula
Source: PeerJ. 2018 May 11;6:e4638. doi: 10.7717/peerj.4638 (PMC5951144; doi:10.7717/peerj.4638)
Supplement: Supplemental Information 1 — Species occurring outside of the Antarctic Convergence (e.g. South Georgia Island) are included. Records are based on Klimpel et al. (2009) and Oguz et al. (2015). Depth ranges of the fish species are taken from Froese & Pauly (2016). Abbreviations: D, Digenea; C, Cestoda; N, Nematoda; A, Acanthocephala; Cr, Crustacea; H, Hirudinea. [file peerj-06-4638-s001.docx]

**Table S1 Parasite taxa of channichthyid species, based on literature data and own studies.** Species occurring outside of the Antarctic Convergence (e.g. South Georgia Island) are included. Records are based on Klimpel et al. (2009) and Oguz et al. (2015). Depth ranges of the fish species are taken from Froese & Pauly, (2016). Abbreviations: D, Digenea; C, Cestoda; N, Nematoda; A, Acanthocephala; Cr, Crustacea; H, Hirudinea.

| **Hosts** | **Depth [m]** | **Taxa** | **Parasite** | **Region** | **Reference** |
| --- | --- | --- | --- | --- | --- |
| *Chaenocephalus aceratus* | 5 - 770 | D | *Gonocerca phycidis* | South Shetland Islands, South Georgia | (Zdzitowiecki, 1979; Palm, Klimpel & Walter, 2007) |
|  |  |  | *Elytrophalloides oatesi* | South Shetland Islands, South Georgia | (Zdzitowiecki, 1979, 1991; Parukhin & Lyadov, 1981) |
|  |  |  | *Genolinea bowersi* | South Shetland Islands, South Georgia | (Zdzitowiecki, 1979, 1991) |
|  |  |  | *Lecithaster macrocotyle* | South Shetland Islands, South Georgia | (Prudhoe & Bray, 1973; Zdzitowiecki, 1979, 1991, 1992, 1997; Palm, Klimpel & Walter, 2007) |
|  |  |  | *Lepidapedon garrardi* | South Shetland Islands | (Zdzitowiecki, 1979) |
|  |  |  | *Macvicaria pennelli* | South Shetland Islands | (Zdzitowiecki, 1979) |
|  |  |  | *Neolebouria antarctica* | South Shetland Islands, South Georgia | (Zdzitowiecki, 1979, 1991; Zdzitowiecki, Pisano & Vacchi, 1993; Zdzitowiecki, 1997; Palm, Klimpel & Walter, 2007) |
|  |  | C | Tetraphyllidea indet. | South Orkney Islands | (Zdzitowiecki, White & Rocka, 1997) |
|  |  |  | *Diphyllobothrium* sp. | South Georgia | (Parukhin & Lyadov, 1981) |
|  |  | A | *Echinorhynchus petrotschenkoi* | South Shetland Islands, South Georgia | (Laskowski & Zdzitowiecki, 2010) |
|  |  |  | *Aspersentis megarhynchus* | South Shetland Islands, Admiralty Bay | (Zdzitowiecki & Rokosz, 1986; Laskowski & Zdzitowiecki, 2010) |
|  |  |  | *Andracantha baylisi* | South Georgia, South Orkneys | (Zdzitowiecki, 1990; Laskowski & Zdzitowiecki, 2010) |
|  |  |  | *Corynosoma bullosum* | South Shetland Islands, South Georgia,  Admiralty Bay, South Orkneys | (Zdzitowiecki, 1990; Palm, Klimpel & Walter, 2007; Laskowski & Zdzitowiecki, 2010) |
|  |  |  | *Corynosoma hamanni* | South Shetland Islands, South Georgia,  Admiralty Bay, South Orkneys | (Zdzitowiecki, 1990; Laskowski & Zdzitowiecki, 2010) |
|  |  |  | *Corynosoma pseudohamanni* | South Shetland, Admiralty Bay,  South Orkneys | (Laskowski & Zdzitowiecki, 2010) |
|  |  |  | *Corynosoma shackletoni* | South Shetland Islands, South Georgia,  Admiralty Bay | (Zdzitowiecki, 1990; Laskowski & Zdzitowiecki, 2010) |
|  |  |  | *Metacanthocephalus dalmori* | Admiralty Bay | (Laskowski & Zdzitowiecki, 2010) |
|  |  |  | *Metacanthocephalus* sp*.* | South Shetland Islands | (Palm, Klimpel & Walter, 2007) |
|  |  | N | *Anisakis* sp. | South Shetland Islands | (Rokicki et al., 2009) |
|  |  |  | *Contracaecum* sp. | South Shetlands Island, South Georgia,  South Orkney Islands, Elephant Island | (Siegel, 1980a; Rokicki et al., 2009) |
|  |  |  | *Contracaecum osculatum* (*s.l.*) | South Shetland Islands | (Palm, Klimpel & Walter, 2007) |
|  |  |  | *Contracaecum radiatum* | South Shetland Islands | (Palm, Klimpel & Walter, 2007) |
|  |  |  | *Pseudoterranova* sp*.* | King George Island | (Dzido et al., 2009) |
|  |  |  | *Pseudoterranova decipiens* (*s.l*.) | South Shetland Islands, Elephant Island,  King George Island | (Parukhin & Lyadov, 1981; Palm, 1999; Rokicki et al., 2009) |
|  |  |  | *Dichelyne fraseri* | South Georgia, South Georgia | (Parukhin & Lyadov, 1981; Zdzitowiecki & Cielecka, 1996) |
|  |  |  | *Ascarophis nototheniae* | South Shetland Islands, South Georgia,  South Orkney Islands | (Rocka, 1999a; Palm, Klimpel & Walter, 2007) |
|  |  | H | *Glyptonotobdella antarctica* | South Orkney Islands, Scotia Sea,  McMurdo Sound, Ross Sea | (Utevsky, 2005) |
|  |  |  | *Trulliobdella bacilliformis* | South Shetland Islands, South Orkney,  South Georgia Islands | (Utevsky, 2005) |
|  |  |  | *Trulliobdella capitis* | South Shetland Islands, South Georgia Islands | (Siegel, 1980b) |
|  |  | Cr | *Eubrachiella antarctica* | Antarctic Peninsula, South Orkney, South Shetland Islands, South Georgia | (Siegel, 1980a) |
| *Chaenodraco wilsoni* | 200 - 800 | D | *Neolebouria antarctica* | South Shetland Islands | this study |
|  |  | C | *Phyllobothrium* sp. | South Shetland Islands | (Wojciechowska, 1993) |
|  |  |  | Diphyllobothriidea indet. | South Shetland Islands | this study |
|  |  | N | *Contracaecum osculatum* (*s.l.*) | South Shetland Islands | this study – new host record |
|  |  |  | *Contracaecum radiatum* | South Shetland Islands | this study – new host record |
|  |  |  | *Contracaecum* sp. | Elephant Island, Shishkov Island | (Rokicki et al., 2009) |
|  |  |  | *Pseudoterranova decipiens* (*s.l.*) | Elephant Island, Shishkov Island | (Rokicki et al., 2009) |
|  |  | H | *Notobdella nototheniae* | South Shetland Islands | this study – new host record |
|  |  |  | *Nototheniobdella sawyeri* | Palmer Coast, South Shetland Islands,  Weddell Sea, Ross Sea | (Utevsky, 2005), this study |
|  |  |  | *Trulliobdella capitis* | South Shetland Islands, South Orkney Islands,  Weddell Sea, Ross Sea | (Kock, Schneppenheim & Siegel, 1984; Utevsky, 2005), this study |
| *Champsocephalus esox* | 50 - 250 | D | *Neolepidapedoides subantarcticus* | Beagle Channel (Tierra del Fuego) | (Jeżewski, Zdzitowiecki & Laskowski, 2014) |
|  |  |  | *Genolinea bowersi* | Beagle Channel (Tierra del Fuego) | (Jeżewski, Zdzitowiecki & Laskowski, 2014) |
|  |  |  | *Derogenes varicus* | Beagle Channel (Tierra del Fuego) | (Jeżewski, Zdzitowiecki & Laskowski, 2014) |
|  |  |  | *Elytrophalloides oatesi* | Beagle Channel (Tierra del Fuego) | (Jeżewski, Zdzitowiecki & Laskowski, 2014) |
|  |  | A | *Heterosentis heteracanthus* | Beagle Channel (Tierra del Fuego) | (Laskowski & Zdzitowiecki, 2009) |
|  |  |  | *Hypoechinorhynchus magellanicus* | Beagle Channel (Tierra del Fuego) | (Laskowski & Zdzitowiecki, 2008, 2009) |
|  |  |  | *Aspersentis johni* | Beagle Channel (Tierra del Fuego) | (Laskowski & Zdzitowiecki, 2009) |
|  |  |  | *Andracantha baylisi* | Beagle Channel (Tierra del Fuego) | (Laskowski, Jeżewski & Zdzitowiecki, 2008; Laskowski & Zdzitowiecki, 2009) |
|  |  |  | *Corynosoma beaglense* | Beagle Channel (Tierra del Fuego) | (Laskowski, Jeżewski & Zdzitowiecki, 2008; Laskowski & Zdzitowiecki, 2009) |
|  |  |  | *Corynosoma evae* | Beagle Channel (Tierra del Fuego) | (Laskowski, Jeżewski & Zdzitowiecki, 2008; Laskowski & Zdzitowiecki, 2009) |
| *Champsocephalus gunnari* | 0 - 700 | D | *Derogenes varicus* | Kerguelen Subregion | (Prudhoe & Bray, 1973) |
|  |  |  | *Gonocerca phycidis* | Kerguelen Subregion, South Shetland Islands | (Prudhoe & Bray, 1973; Zdzitowiecki, 1991) |
|  |  |  | *Elytrophalloides oatesi* | South Georgia Islands | (Zdzitowiecki, 1979) |
|  |  |  | *Lecithaster macrocotyle* | Kerguelen Subregion | (Prudhoe & Bray, 1973; Parukhin & Lyadov, 1981) |
|  |  |  | *Lecithophyllum champsocephali* | South Shetland Islands | (Zdzitowiecki, 2002) |
|  |  |  | *Macvicaria georgiana* | South Shetland Islands | this study – new host record |
|  |  |  | *Macvicaria pennelli* | South Shetland Islands | (Zdzitowiecki, 1991) |
|  |  |  | *Neolebouria antarctica* | South Shetland Islands, South Georgia Island,  Elephant Island, Elephant Island | (Zdzitowiecki, 1991) |
|  |  |  | *Caudotestis kerguelensis* | Kerguelen Subregion | (Prudhoe & Bray, 1973) |
|  |  | C | Diphyllobothriidea indet. | South Shetland Islands | this study |
|  |  |  | Tetraphyllidae indet. | Kerguelen Subregion, Heard Island | (Parukhin & Lyadov, 1981; Wojciechowska, Pisano & Zdzitowiecki, 1995) |
|  |  |  | *Bothriocephalus antarcticus* | Heard Island | (Wojciechowska, Pisano & Zdzitowiecki, 1995) |
|  |  |  | *Phyllobothrium* sp. | Kerguelen Subregion | (Parukhin & Lyadov, 1981) |
|  |  | A | *Metacanthocephalus rennicki* | Kerguelen Subregion | (Parukhin & Lyadov, 1981) |
|  |  |  | *Corynosoma hamanni* | South Georgia Islands | (Parukhin & Lyadov, 1981) |
|  |  |  | *Corynosoma pseudohamanni* | South Shetland Islands | (Zdzitowiecki, 1986) |
|  |  |  | *Metacanthocephalus dalmori* | South Shetland Islands | (Zdzitowiecki, 1986) |
|  |  |  | *Metacanthocephalus johnstoni* | South Shetland Islands | (Zdzitowiecki, 1986) |
|  |  | N | *Anisakis* sp*.* | Kerguelen Subregion | (Lyadov, Parukhin & Mironova, 1981; Parukhin & Lyadov, 1981) |
|  |  |  | *Contracaecum osculatum* (*s.l*.) | South Shetland Islands | this study – new host record |
|  |  |  | *Contracaecum radiatum* | South Shetland Islands | this study – new host record |
|  |  |  | *Contracaecum sp.* | South Shetland Islands, South Georgia,  South Orkney Islands, Elephant Island | (Siegel, 1980a; Rokicki et al., 2009) |
|  |  |  | *Pseudoterranova decipiens* (*s.l.*) | King Georgie Island | (Rokicki et al., 2009) |
|  |  |  | *Dichelyne fraseri* | Kerguelen Subregion | (Parukhin & Lyadov, 1981) |
|  |  |  | *Ascarophis morrhuae* | South Georgia Islands | (Parukhin & Lyadov, 1981) |
|  |  |  | *Ascarophis draconi* | South Chile | (Munoz & George-Nascimento, 2007) |
|  |  | H | *Trulliobdella bacilliformis* | Bouvet Island, South Shetland Islands,  South Orkney Islands, Kerguelen Subregion | (Utevsky, 2005) |
|  |  |  | *Trulliobdella capitis* | South Shetland Islands, South Georgia Island,  Ross Sea, Weddell Sea, South Orkney Islands, Wilkes Land | (Siegel, 1980b; Utevsky, 2005) |
| *Channichthys rhinoceratus* | 1 - 750 | D | *Elytrophalloides oatesi* | Kerguelen Subregion | (Lyadov, Parukhin & Mironova, 1981; Parukhin & Lyadov, 1981) |
|  |  |  | *Derogenes varicus* | Heard Island | (Zdzitowiecki & Pisano, 1996) |
|  |  |  | *Gonocerca phycidis* | Kerguelen Island | (Parukhin & Lyadov, 1981) |
|  |  |  | *Lecithaster macrocotyle* | Kerguelen Island | (Parukhin & Lyadov, 1981) |
|  |  |  | *Lecithaster micropsi* | Heard Island | (Parukhin & Lyadov, 1981) |
|  |  | C | Tetraphyllidea indet. | Heard Island Plateau | (Wojciechowska, Pisano & Zdzitowiecki, 1995) |
|  |  |  | *Bothriocephalus antarcticus* | Heard Island Plateau | (Wojciechowska, Pisano & Zdzitowiecki, 1995) |
|  |  | A | *Corynosoma bullosum* | Heard Island | (Zdzitowiecki & Pisano, 1996) |
|  |  |  | *Corynosoma hamanni* | Kerguelen Island | (Lyadov, Parukhin & Mironova, 1981) |
|  |  | N | *Anisakis* sp. | Kerguelen Island | (Parukhin & Lyadov, 1981) |
|  |  |  | *Contracaecum* sp*.* | Kerguelen Island | (Parukhin & Lyadov, 1981) |
|  |  |  | *Dichelyne fraseri* | Kerguelen Island | (Parukhin & Lyadov, 1981) |
|  |  | Cr | *Eubrachiella gaini gaini* | Kerguelen Island, Heard Island | (Lyadov, Parukhin & Mironova, 1981; Rohde et al., 1998) |
|  |  |  | *Crustacea* indet. | Heard Island | (Rohde, Hayward & Heap, 1995) |
| *Chionodraco hamatus* | 4 - 600 | D | *Derogenes johnstoni* | Adélie Land, Weddell Sea, Ross Sea | (Zdzitowiecki, 1993, 2002; Santoro et al., 2014) |
|  |  |  | *Elytrophalloides oatesi* | Adélie Land, Ross Sea | (Zdzitowiecki, 2001; Santoro et al., 2014) |
|  |  |  | *Genolinea bowersi* | Adélie Land, Ross Sea | (Zdzitowiecki, 2001; Santoro et al., 2014) |
|  |  |  | *Gonocerca phycidis* | Ross Sea | (Santoro et al., 2014) |
|  |  |  | *Lepidapedon garrardi* | Ross Sea | (Santoro et al., 2014) |
|  |  |  | *Neolebouria terranovaensis* | Adélie Land, Ross Sea | (Zdzitowiecki, Pisano & Vacchi, 1993; Zdzitowiecki, 2002; Santoro et al., 2014) |
|  |  |  | *Macvicaria georgiana* | Ross Sea | (Santoro et al., 2014) |
|  |  | C | Tetraphyllidea indet. | Adélie Land, Ross Sea | (Zdzitowiecki, 2001; Santoro et al., 2014) |
|  |  |  | Diphyllobothriidae indet. | Adélie Land, Ross Sea | (Santoro et al., 2014) |
|  |  | A | *Corynosoma pseudohamanni* | Ross Sea | (Santoro et al., 2014) |
|  |  |  | *Corynosoma hamanni* | Ross Sea | (Santoro et al., 2014) |
|  |  |  | *Metacanthocephalus* sp. | Ross Sea | (Zdzitowiecki, Palladino & Vacchi, 1999) |
|  |  |  | *Metacanthocephalus campbelli* | Adélie Land, Ross Sea | (Zdzitowiecki, Palladino & Vacchi, 1999) |
|  |  |  | *Metacanthocephalus rennicki* | Ross Sea | (Santoro et al., 2014) |
|  |  | N | *Contracaecum* sp. | Elephant Island, Shishkov Islands | (Zdzitowiecki et al., 1998) |
|  |  |  | *Contracaecum osculatum* (s.l.) | Ross Sea | (Santoro et al., 2013) |
|  |  |  | *Contracaecum osculatum* D | Ross Sea | (Santoro et al., 2014) |
|  |  |  | *Contracaecum osculatum* E | Ross Sea | (Santoro et al., 2014) |
|  |  |  | *Contracaecum radiatum* | Ross Sea | (Santoro et al., 2014) |
|  |  |  | *Pseudoterranova decipiens* (*s.l*.) | Adélie Land, Weddell Sea | (Palm, 1999) |
|  |  |  | *Ascarophis nototheniae* | Adélie Land, Ross Sea | (Santoro et al., 2014) |
|  |  | H | *Nototheniobdella sawyeri* | Palmer Coast, South Shetland Islands,  Weddell Sea, Clarie Coast | (Utevsky, 2005; Santoro et al., 2014) |
|  |  |  | *Trulliobdella capitis* | Bouvet Island, South Shetland Islands,  South Orkney Islands, Weddell Sea, Wilkes Land,  Argentine Island, Ross Sea | (Utevsky, 2005; Santoro et al., 2014) |
|  |  |  | *Cryobdella antarctica* | Ross Sea | (Santoro et al., 2014) |
|  |  | Cr | *Eubrachiella gaini* | Ross Sea | (Santoro et al., 2014) |
|  |  |  | *Caecognathia calva* | Ross Sea | (Santoro et al., 2014) |
| *Chionodraco myersi* | 200 - 800 | C | Tetraphyllidea indet. | Weddell Sea | (Rocka, 1999a) |
|  |  | N | *Contracaecum osculatum* (*s.l.*) | Weddell Sea | (Klöser et al., 1992) |
|  |  |  | *Contracaecum radiatum* | Weddell Sea | (Klöser et al., 1992) |
| *Neopagetopsis ionah* | 20 - 900 | D | *Neolebouria antarctica* | South Shetland Islands, Admiralty Bay,  King George Island, Elephant Island | (Zdzitowiecki, 1993, 2002) |
|  |  | C | *Phyllobothrium* sp. | South Shetland Islands | (Wojciechowska, 1993) |
|  |  |  | Diphyllobothriidea indet. | South Shetland Islands | this study |
|  |  | N | *Contracaecum osculatum (s.l.)* | South Shetland Islands | this study – new host record |
|  |  |  | *Contracaecum radiatum* | South Shetland Islands | this study – new host record |
|  |  | H | *Nototheniobdella sawyeri* | Palmer Coast, South Shetland Islands,  Weddell Sea, Scott Coast, Ross Sea | (Utevsky, 2005) |
|  |  |  | *Trulliobdella capittis* | Bouvet Island, South Shetland Islands,  South Orkney Islands, Weddell Sea, Wilkes Land,  Argentine Island, Ross Sea | (Utevsky, 2005) |
| *Pagetopsis macropterus* | 5 - 655 | D | *Elytrophalloides oatesi* |  | (Prudhoe & Bray, 1973) |
|  |  | N | *Contracaecum osculatum (s.l.)* | South Shetland Islands | this study – new host record |
|  |  |  | *Contracaecum radiatum* | South Shetland Islands | this study – new host record |
|  |  |  | *Contracaecum sp.* | Elephant Island, Shishkov Islands | (Rokicki et al., 2009) |
|  |  | H | *Nototheniobdella sawyeri* | South Shetland Islands | this study |
| *Pseudochaenichthys georgianus* | 0 - 475 | D | *Gonocerca phycidis* | South Georgia Island, South Shetland Islands | (Zdzitowiecki, 1979, 1991, 2002), this study |
|  |  |  | *Elytrophalloides oatesi* | South Georgia Island | (Zdzitowiecki, 1979), this study |
|  |  |  | *Lecithaster macrocotyle* | South Georgia Island | (Zdzitowiecki, 1991) |
|  |  |  | *Lecithaster micropsi* | Shag Rocks | (Zdzitowiecki, 1997) |
|  |  |  | *Neolebouria antarctica* | South Shetland Islands, South Georgia Island,  Elephant Island | (Zdzitowiecki, Pisano & Vacchi, 1993), this study |
|  |  | C | *Phyllobothrium* sp. | South Shetland Islands , South Georgia Islands | (Wojciechowska, 1993) |
|  |  |  | Diphyllobothriidea indet. | South Shetland Islands | this study |
|  |  |  | Tetraphyllidae indet. | South Shetland Islands | this study |
|  |  | N | *Anisakis* sp. | Elephant Island, Shishkov Island | (Rokicki et al., 2009) |
|  |  |  | *Contracaecum osculatum* (*s.l.*) | South Shetland Islands | this study – new host record |
|  |  |  | *Contracaecum radiatum* | South Shetland Islands | this study – new host record |
|  |  |  | *Contracaecum* sp. | South Georgia Island, South Orkney Islands,  South Shetlands Islands, Elephant Island | (Siegel, 1980b) |
|  |  |  | *Pseudoterranova decipiens* (*s.l.*) | South Shetland Islands | (Palm, 1999); this study |
|  |  |  | *Dichelyne fraseri* | South Georgia | (Parukhin & Lyadov, 1981) |
|  |  |  | *Ascarophis nototheniae* | South Shetland Islands | this study – new host record |
|  |  | A | *Corynosoma bullosum* | South Shetland Islands | this study – new host record |
|  |  | H | *Trulliobdella bacilliformis* | Bouvet Island, South Shetland Islands,  South Georgia Islands, South Orkney Islands,  Prince Edward Island, Kerguelen Subregion | (Utevsky, 2005) |
|  |  |  | *Trulliobdella capitis* | South Georgia Island, South Shetland Islands,  South Orkney Islands, Bouvet Island, Weddell Sea,  Wilkes Land, Argentine Island, Ross Sea | (Siegel, 1980b; Utevsky, 2005) |
|  |  |  | *Nototheniobdella sawyeri* | South Shetland Islands | this study – new host record |
|  |  | Cr | *Eubrachiella antarctica* | South Shetland | this study – new host record |
|  |  |  | *Eubrachiella gaini gaini* | South Shetland Islands, South Orkneys,  South Georgia | (Sosinski & Janusz, 1986) |
| *Chionodraco rastrospinosus* | 0 - 1000 | D | *Gonocerca phycidis* | South Shetland Islands | (Zdzitowiecki, 1991) |
|  |  |  | *Elytrophalloides oatesi* | South Shetland Islands | (Zdzitowiecki, 1991) |
|  |  |  | *Genolinea bowersi* | South Shetland Islands | (Zdzitowiecki, 1988) |
|  |  |  | *Lecithaster macrocotyle* | South Shetland Islands | (Zdzitowiecki, 1991, 2002) |
|  |  |  | *Lepidapedon garrardi* | South Shetland Islands | (Zdzitowiecki, 1991) |
|  |  |  | *Neolebouria antarctica* | South Shetland Islands, King George Island,  Elephant Island, South Georgia | (Zdzitowiecki, 1991; Zdzitowiecki, Pisano & Vacchi, 1993) |
|  |  | A | *Corynosoma bullosum* | South Shetland Islands | (Zdzitowiecki, 1986) |
|  |  |  | *Corynosoma pseudohamanni* | South Shetland Islands | (Zdzitowiecki, 1990) |
|  |  |  | *Metacanthocephalus dalmori* | South Shetland Islands | (Zdzitowiecki, 1990) |
| *Cryodraco antarcticus* |  | D | *Elytrophalloides oatesi* | South Shetland Islands, South Georgia | (Zdzitowiecki, 1991) |
|  |  |  | *Genolinea bowersi* | South Shetland Islands | (Zdzitowiecki, 1991) |
|  |  |  | *Glomericirrus macrouri* | South Shetland Islands, Weddell Sea | (Zdzitowiecki, 1991, 1997) |
|  |  |  | *Gonocerca phycidis* | South Shetland Islands | (Zdzitowiecki, 1991) |
|  |  |  | *Lecithaster macrocotyle* | South Shetland Islands | (Zdzitowiecki, 1991) |
|  |  |  | *Macvicaria georgiana* | Weddell Sea | (Zdzitowiecki & Cielecka, 1997) |
|  |  |  | *Neolebouria antarctica* | South Shetland Islands | (Zdzitowiecki, 1991; Zdzitowiecki, Pisano & Vacchi, 1993) |
|  |  | C | Tetraohyllidea indet. | Weddell Sea | (Rocka, 1999a) |
|  |  | N | *Ascarophis nototheniae* | South Shetland Islands, Ross Sea | (Rocka, 1999b) |
|  |  |  | *Contracaecum osculatum* (*s.l*.) | Weddell Sea | (Klöser et al., 1992) |
|  |  |  | *Contracaecum radiatum* | Weddell Sea | (Klöser et al., 1992) |
|  |  |  | *Pseudoterranova decipiens* (*s.l.*) | Weddell Sea | (Palm et al., 1994; Palm, 1999) |
|  |  |  | *Corynosoma bullosum* | South Shetland Islands | (Zdzitowiecki, 1990) |
|  |  |  | *Corynosoma hamanni* | South Shetland Islands | (Zdzitowiecki, 1990) |
|  |  |  | *Corynosoma pseudohamanni* | South Shetland Islands | (Zdzitowiecki, 1990) |
|  |  |  | *Echinorhynchus petrotschenkoi* | South Shetland Islands | (Zdzitowiecki, 1990) |
|  |  |  | *Metacanthocephalus dalmori* | South Shetland Islands | (Zdzitowiecki, 1990) |

**References**

Dzido J., Kijewska A., Rokicka M., Świątalska-Koseda A., Rokicki J. 2009. Report on anisakid nematodes in polar regions – Preliminary results. *Polar Science* 3:207–211. DOI: 10.1016/j.polar.2009.08.003.

Froese R., Pauly D. 2018. FishBase. *Available at* *http:\\www.fishbase.org*

Jeżewski W., Zdzitowiecki K., Laskowski Z. 2014. Digenea in notothenioid fish in the Beagle Channel (Magellanic sub-region, sub-Antarctica). *Acta Parasitologica* 59:42–49. DOI: 10.2478/s11686-014-0208-4.

Klimpel S., Busch MW., Kellermanns E., Kleinertz S., Palm HW. 2009. *Metazoan Deep Sea Fish Parasites*. Solingen: Verlag Natur & Wissenschaft.

Klöser H., Plötz J., Palm H., Bartsch A., Hubold G. 1992. Adjustment of anisakid nematode life cycles to the high Antarctic food web as shown by *Contracaecum radiatum* and *C. osculatum* in the Weddell Sea. *Antarctic Science* 4:171–178.

Kock KH., Schneppenheim R., Siegel V. 1984. A contribution to the fish fauna of the Weddell Sea. *Archiv für Fischereiwissenschaft* 34:103–120.

Laskowski Z., Jeżewski W., Zdzitowiecki K. 2008. Cystacanths of Acanthocephala in notothenioid fish from the Beagle Channel (sub-Antarctica). *Systematic Parasitology* 70:107–117.

Laskowski Z., Zdzitowiecki K. 2008. New morphological data on the acanthocephalan *Hypoechinorhynchus magellanicus* Szidat, 1950 (Palaeacanthocephala: Arhythmacanthidae). *Systematic parasitology* 69:179–183.

Laskowski Z., Zdzitowiecki K. 2009. Occurrence of acanthocephalans in notothenioid fishes in the Beagle Channel (Magellanic sub-region, sub-Antarctic). *Polish Polar Research* 30:179–186.

Laskowski Z., Zdzitowiecki K. 2010. Contribution to the knowledge of the infection with Acanthocephala of a predatory Antarctic ice-fish *Chaenocephalus aceratus*. *Polish Polar Research* 31:303–308.

Lyadov VN., Parukhin AM., Mironova VA. 1981. Helminth fauna of fishes of the family Chaenichthyidae from the region of Kerguelen Islands. *Zoologicheskii zhurnal* 60:142–144.

Munoz G., George-Nascimento M. 2007. Two new species of *Ascarophis* (Nematoda: Cystidicolidae) in marine fishes from Chile. *Journal of Parasitology* 93:1178–1188.

Oguz MC., Tepe Y., Belk MC., Heckmann RA., Aslan B., Gurgen M., Bray RA., Akgul U. 2015. Metazoan Parasites of Antarctic Fishes. *Turkish Journal of Parasitology* 39:174–178. DOI: 10.5152/tpd.2015.3661.

Palm HW. 1999. Ecology of *Pseudoterranova decipiens* (Krabbe, 1878)(Nematoda: Anisakidae) from Antarctic waters. *Parasitology Research* 85:638–646.

Palm H., Andersen K., Klöser H., Plötz J. 1994. Occurrence of *Pseudoterranova decipiens* (Nematoda) in fish from the southeastern Weddell Sea (Antarctic). *Polar Biology* 14:539–544.

Palm HW., Klimpel S., Walter T. 2007. Demersal fish parasite fauna around the South Shetland Islands: high species richness and low host specificity in deep Antarctic waters. *Polar Biology* 30:1513–1522.

Parukhin AM., Lyadov VN. 1981. Parasitofauna of Notothenioidei from waters of the Atlantic and Indian Oceans. *Vestnik Zoologii* 3:90–94.

Prudhoe S., Bray RA. 1973. *Digenetic trematodes from fishes*. BANZ Antarctic Research Expedition Report Series B 8:195–225.

Rocka A. 1999a. The tetraphyllidean cercoids from teleosts occurring in the Weddell Sea (Antarctic). *Acta Parasitologica* 44:115–118.

Rocka A. 1999b. Biometrical variability and occurrence of *Ascarophis nototheniae* (Nematoda, Cystidicolidae), a parasitic nematode of Antarctic and subantarctic fishes. *Acta Parasitologica* 44:188–192.

Rohde K., Hayward C., Heap M. 1995. Aspects of the ecology of metazoan ectoparasites of marine fishes. *International Journal for parasitology* 25:945–970.

Rohde K., Ho J-S., Smales L., Williams R. 1998. Parasites of Antarctic fishes: Monogenea, Copepoda and Acanthocephala. *Marine and Freshwater Research* 49:121–125.

Rokicki J., Rodjuk G., Zdzitowiecki K., Laskowski Z. 2009. Larval ascaridoid nematodes (Anisakidae) in fish from the South Shetland Islands (Southern Ocean). *Polish Polar Research* 30:49–58.

Santoro M., Mattiucci S., Cipriani P., Bellisario B., Romanelli F., Cimmaruta R., Nascetti G. 2014. Parasite Communities of Icefish (Chionodraco hamatus) in the Ross Sea (Antarctica): Influence of the Host Sex on the Helminth Infracommunity Structure. *PLOS ONE* 9:e88876. DOI: 10.1371/journal.pone.0088876.

Santoro M., Mattiucci S., Work T., Cimmaruta R., Nardi V., Cipriani P., Bellisario B., Nascetti G. 2013. Parasitic infection by larval helminths in Antarctic fishes: pathological changes and impact on the host body condition index. *Diseases of Aquatic Organisms* 105:139–48.

Siegel V. 1980a. Parasite tags for some Antarctic channichthyid fish. *Archiv fur Fischereiwissenschaft* 31:97–103.

Siegel V. 1980b. Quantitative investigations on parasites of antarctic channichthyid and nototheniid fishes. *Meeresforschung* 28:146–156.

Sosinski J., Janusz J. 1986. The occurrence of the parasite *Eubrachiella gaini* (Quidor, 1913) in Antarctic fishes of the family Chaenichthyidae. *Acta Ichthyologica et Piscatoria* 16:87–105.

Utevsky AY. 2005. An identification key to Antarctic fish leeches (Hirudinea: Piscicolidae). *Ukrainian Antarctic Journal* 3:135–144.

Wojciechowska A. 1993. The tetraphyllidean and tetrabothriid cercoids from Antarctic bony fishes. II. Occurrence of cercoids in various fish species. *Acta Parasitologica* 38:113–118.

Wojciechowska A., Pisano E., Zdzitowiecki K. 1995. Cestodes in fishes at tha Heard Island (Subantarctic). *Polish Polar Research* 16:205–212.

Zdzitowiecki K. 1979. Digenetic trematodes in alimentary tracts of fishes of South Georgia and South Shetlands (Antarctica). *Acta Ichthyologica et Piscatoria* 9:15–30.

Zdzitowiecki K. 1986. Prevalence of Acanthocephalans in Fishes of South Shetlands (Antarctic. III. Metacanthocephalus Johnstoni Zdzitowiecki, 1983, M. Dalmori Zdzitowiecki, 1983 and Notes on Other Species; General Conclusions. *Acta Parasitologica Polonica* 31:125–141.

Zdzitowiecki K. 1988. Occurrence of digenetic trematodes in fishes off South Shetlands (Antarctic). *Acta Parasitologica Polonica* 33.

Zdzitowiecki K. 1990. Occurrence of acanthocephalans in fishes of the open sea off the South Shetlands and South Georgia (Antarctic). *Acta Parasitologica Polonica* 35:131–141.

Zdzitowiecki K. 1991. Occurrence of digeneans in open sea fishes off the South Shetland Islands and South Georgia, and a list of fish digeneans in the Antarctic. *Polish Polar Research* 12:55–72.

Zdzitowiecki K. 1992. Antarctic representatives of the genus Lecithaster Luhe, 1901 [Digenea, Hemiuridae], with the description of a new species. *Acta Parasitologica* 37:57–63.

Zdzitowiecki K. 1993. A contribution to the morphology of the Antarctic fish lepocreadiid digeneas, with a description of a new genus. *Acta Parasitologica* 38:109–112.

Zdzitowiecki K. 1997. *Antarctic Digenea, parasites of fishes*. Koenigstein: Koeltz Scientific Books.

Zdzitowiecki K. 2001. New data on the occurrence of fish endoparasitic worms off Adelie Land, Antarctica. *Polish Polar Research* 22:159–165.

Zdzitowiecki K. 2002. Occurrence of Digenea in fishes of the family Channichthyidae in the Weddell Sea and other sub-continental areas of the Antarctica. *Acta Parasitologica* 47:159–162.

Zdzitowiecki K., Cielecka D. 1996. Morphology and occurrence of *Dichelyne* [*Cucullanellus*] *fraseri* [Baylis, 1929], a parasitic nematode of Antarctic and sub-Antarctic fishes. *Acta Parasitologica* 41:30–37.

Zdzitowiecki K., Cielecka D. 1997. Digenea of fishes of the Weddell Sea. II. The genus Macvicaria (Opecoelidae). *Acta Parasitologica* 42:77–83.

Zdzitowiecki K., Palladino S., Vacchi M. 1999. Acanthocephala found in fish in the Terra Nova Bay (Ross Sea, Antarctica). *Polish Polar Research* 20:59–63.

Zdzitowiecki K., Pisano E. 1996. New records of Digenea infection elasmobranch and teleost fish off Heard Island (Kerguelen sub-region, sub-Antarctic). *Archive of Fishery and Marine Research* 43:265–272.

Zdzitowiecki K., Pisano E., Vacchi M. 1993. Antarctic representatives of the genus *Neolebouria* Gibson, 1976 [Digenea, Opecoelidae] with description of one new species. *Acta Parasitologica* 38:11–14.

Zdzitowiecki K., Rocka A., Pisano E., Ozouf-Costaz C. 1998. A list of fish parasitic worms collected off Adelie Land [Antarctic]. *Acta Parasitologica* 43:71–74.

Zdzitowiecki K., Rokosz B. 1986. Prevalence of acanthocephalans in fishes of South Shetlands (Antarctic). II. *Aspersentis austrinus* Van Cleave, 1929 and remarks on the validity of Heteracanthocephalus hureaui Dollfus, 1965. *Acta Parasitologica Polonica* 30:161–171.

Zdzitowiecki K., White MG., Rocka A. 1997. Digenean, monogenean and cestode infection of inshore fish at the South Orkney Islands. *Acta Parasitologica* 42:18–22.
