# Supplementary material for: Lighten up the dark: metazoan parasites as indicators for the ecology of Antarctic crocodile icefish (Channichthyidae) from the north-west Antarctic Peninsula
Source: PeerJ. 2018 May 11;6:e4638. doi: 10.7717/peerj.4638 (PMC5951144; doi:10.7717/peerj.4638)
Supplement: Supplemental Information 2 — n = sample size, TL = total length, TW = total weight, SW = slaughter weight. Arithmetric mean and standard deviation are given. Median, minimum and maximum are given below. K = condition factor, HSI = hepatosomatic index. [file peerj-06-4638-s002.docx]

**S2:** **Morphometric data on the host species investigated.** n = sample size, TL = total length, TW = total weight, SW = slaughter weight. Arithmetric mean and standard deviation are given. Median, minimum and maximum are given below. K = condition factor, HSI = hepatosomatic index.

| **Species** | **TL [cm]** | **TG [g]** | **SW [g]** | **K** | **HSI** |  |
| --- | --- | --- | --- | --- | --- | --- |
| ***C. wilsoni*** | 29.9 +/-4.4 | 224.4 +/- 85.8 | 170.1+/-67.2 | 0.58 | 2.7 |  |
| **(n = 33)** | 32.1 | 249.6 | 184.5 |  |  |  |
|  | 15.4 - 35.1 | 16.4 - 346.9 | 11.2 - 271.1 |  |  |  |
| ***C. gunnari*** | 29.8 +/- 7.0 | 196.3 +/- 120.6 | 153.7 +/- 92.9 | 0.49 | 1.8 |  |
| **(n = 25)** | 31.5 | 169.7 | 122.4 |  |  |  |
|  | 13.2 - 36.5 | 12.8 - 412 | 9.6 - 319 |  |  |  |
| ***N. ionah*** | 52.3 +/- 2.1 | 1147.0 +/- 88.4 | 873.6 +/- 67.3 | 0.61 | 2.9 |  |
| **(n = 3)** | 51.4 | 1143.0 | 839.3 |  |  |  |
|  | 50.8 - 54.7 | 1060.9 - 1237.5 | 830.3 - 951.1 |  |  |  |
| ***P. macropterus*** | 22.8 +/- 3.0 | 97.1 +/- 38.2 | 71.1 +/- 27.7 | 0.59 | 2.3 |  |
| **(n = 4)** | 23.5 | 90.9 | 65.9 |  |  |  |
|  | 18.6 - 25.4 | 57.4 - 149.3 | 43.4 - 109.3 |  |  |  |
| ***P. georgianus*** | 36.7 +/- 7.3 | 580.8 +/- 444.4 | 396.7 +/- 257.6 | 0.68 | 2.9 |  |
| **(n = 15)** | 35.1 | 406.4 | 306.4 |  |  |  |
|  | 22.3 - 47.2 | 65.0 - 1639.6 | 48.2 - 882.1 |  |  |  |
