# Supplementary material for: Lighten up the dark: metazoan parasites as indicators for the ecology of Antarctic crocodile icefish (Channichthyidae) from the north-west Antarctic Peninsula
Source: PeerJ. 2018 May 11;6:e4638. doi: 10.7717/peerj.4638 (PMC5951144; doi:10.7717/peerj.4638)
Supplement: Supplemental Information 3 — Nematodes subjected to molecular analyses and identity matching with the NCBI data set (GenBank). ID = Sample code: Species initials_Host-No._Nematode-No., Organism = Species of highest identity in GenBank, % Identity: Pairwise identity of query and organism. [file peerj-06-4638-s003.docx]

**S3:** **Results of molecular species identification.** Nematodes subjected to molecular analyses and GenBank BLASTn results including the organism with the highest sequence identit. ID = identification of parasite, Organism = species of highest identity in GenBank, % Identity: Pairwise identity of query and organism, E-value = GenBank significance value, Accession = GenBank accession number.

| **Host** | **ID** | **Length** | **Organism** | **% Identity** |  | **E Value** | **Accession** |
| --- | --- | --- | --- | --- | --- | --- | --- |
| ***C. wilsoni*** | Cw_19_2.2b | 466 | *Contracaecum* cf. *osculatum* D | 98.50 |  | 0 | EU477207 |
|  | Cw_22_2.1 | 452 | *Contracaecum* cf. *osculatum* D | 98.90 |  | 0 | KC412223 |
|  | Cw_22_2b | 469 | *Contracaecum* cf. *osculatum* D | 98.50 |  | 0 | KC412227 |
|  | Cw_21_2 | 468 | *Contracaecum* cf. *osculatum* D | 98.70 |  | 0 | KF771354 |
|  | Cw_24_2.1 | 451 | *Contracaecum* cf. *osculatum* D | 98.70 |  | 0 | KF771354 |
|  | Cw_18_2.1 | 417 | *Contracaecum* cf. *osculatum* D | 99.00 |  | 0 | KF771361 |
|  | Cw_19_2.2a | 475 | *Contracaecum* cf. *osculatum* D | 98.70 |  | 0 | KF771361 |
|  | Cw_20_2 | 469 | *Contracaecum* cf. *osculatum* D | 99.40 |  | 0 | KF771361 |
|  | Cw_23_2.2a | 467 | *Contracaecum* cf. *osculatum* D | 98.90 |  | 0 | KT285806 |
|  | Cw_14_2.4 | 470 | *Contracaecum* cf. *osculatum* E | 99.40 |  | 0 | EU477205 |
|  | Cw_23_2.2b | 471 | *Contracaecum* cf. *osculatum* E | 98.50 |  | 0 | KF771355 |
|  | Cw_14_2c | 438 | *Contracaecum* cf. *osculatum* E | 98.90 |  | 0 | KT285805 |
|  | Cw_22_2e | 457 | *Contracaecum* cf. *osculatum* E | 99.30 |  | 0 | KT285805 |
|  | Cw_23_2 | 438 | *Contracaecum* cf. *osculatum* E | 98.90 |  | 0 | KT285809 |
|  | Cw_18_2.2 | 469 | *Contracaecum osculatum* | 99.10 |  | 0 | KF718939 |
|  | Cw_19_2.3 | 444 | *Contracaecum osculatum* | 98.50 |  | 0 | KF718939 |
|  | Cw_20_5 | 458 | *Contracaecum radiatum* | 98.30 |  | 0 | EU477210 |
|  | Cw_12_3.2 | 458 | *Contracaecum radiatum* | 99.10 |  | 0 | EU477210 |
|  | Cw_20_3.1 | 460 | *Contracaecum radiatum* | 98.90 |  | 0 | EU477210 |
|  | Cw_29_3 | 444 | *Contracaecum radiatum* | 97.70 |  | 0 | EU477210 |
|  | Cw_31_3.1 | 464 | *Contracaecum radiatum* | 97.80 |  | 0 | EU477210 |
|  | Cw_32_4 | 458 | *Contracaecum radiatum* | 98.90 |  | 0 | EU477210 |
|  | Cw_4_5 | 448 | *Contracaecum radiatum* | 98.10 |  | 0 | EU477210 |
|  | Cw_12_5.3 | 546 | *Contracaecum* sp. | 94.10 |  | 0 | KF718911 |
|  | Cw_22_3 | 415 | *Contracaecum* sp. | 93.60 |  | 1.80E-173 | KF718911 |
|  | Cw_19_5 | 451 | *Contracaecum* sp. | 93.10 |  | 0 | KF718924 |
|  | Cw_20_3 | 454 | *Parascaris equorum* | 85.10 |  | 3.21E-126 | AP017696 |
|  | Cw_22_2.1c | 541 | *Parascaris equorum* | 84.80 |  | 4.01E-150 | AP017696 |
|  | Cw_22_2c | 501 | *Parascaris equorum* | 84.20 |  | 6.85E-133 | AP017696 |
| ***C. gunnari*** | Cg_10_2.1 | 429 | *Contracaecum* aff. *multipapillatum* A | 86.50 |  | 8.93E-127 | EU852338 |
|  | Cg_20_2a | 429 | *Contracaecum* aff. *multipapillatum* A | 86.50 |  | 3.21E-126 | EU852338 |
|  | Cg_12_2.1 | 371 | *Contracaecum* aff. *multipapillatum* A | 86.90 |  | 1.53E-109 | EU852340 |
|  | Cg_16_2 | 469 | *Contracaecum* cf. *osculatum* D | 99.40 |  | 0 | KF771354 |
|  | Cg_4_2 | 469 | *Contracaecum* cf. *osculatum* E | 99.10 |  | 0 | KT285805 |
|  | Cg_4_2.1 | 469 | *Contracaecum* cf. *osculatum* E | 99.40 |  | 0 | KT285805 |
|  | Cg_4_2.1b | 457 | *Contracaecum* cf. *osculatum* E | 99.30 |  | 0 | KT285805 |
|  | Cg_4_2.1c | 456 | *Contracaecum* cf. *osculatum* E | 99.10 |  | 0 | KT285805 |
|  | Cg_2_2.1 | 471 | *Contracaecum osculatum* | 98.70 |  | 0 | KF718940 |
|  | Cg_22_3 | 469 | *Contracaecum radiatum* | 97.90 |  | 0 | EU477210 |
|  | Cg_16_3 | 546 | *Contracaecum* sp. | 98.50 |  | 0 | KF718906 |
|  | Cg_2_5 | 543 | *Contracaecum* sp. | 99.20 |  | 0 | KF718906 |
|  | Cg_13_3 | 542 | *Contracaecum* sp. | 99.00 |  | 0 | KF718914 |
|  | Cg_25_2 | 542 | *Contracaecum* sp. | 99.00 |  | 0 | KF718914 |
|  | Cg_20_5a | 541 | *Contracaecum* sp. | 99.00 |  | 0 | KF718924 |
|  | Cg_25_2.1 | 536 | *Contracaecum* sp. | 92.60 |  | 0 | KF718927 |
|  | Cg_11_2.1 | 524 | *Parascaris equorum* | 84.90 |  | 1.12E-145 | AP017696 |
|  | Cg_15_3 | 507 | *Parascaris equorum* | 84.70 |  | 1.46E-139 | AP017696 |
|  | Cg_18_4 | 544 | *Parascaris equorum* | 84.20 |  | 4.04E-145 | AP017696 |
|  | Cg_20_2b | 524 | *Parascaris equorum* | 84.90 |  | 1.12E-145 | AP017696 |
|  | Cg_20_2c | 541 | *Parascaris equorum* | 84.30 |  | 4.04E-145 | AP017696 |
|  | Cg_20_5b | 507 | *Parascaris equorum* | 84.20 |  | 5.30E-134 | AP017696 |
|  | Cg_21_3 | 532 | *Parascaris equorum* | 84.70 |  | 1.12E-145 | AP017696 |
|  | Cg_23_3.2a | 559 | *Parascaris equorum* | 84.80 |  | 3.98E-155 | AP017696 |
|  | Cg_23_3.2b | 530 | *Parascaris equorum* | 84.90 |  | 2.41E-147 | AP017696 |
|  | Cg_23_3.2c | 539 | *Parascaris equorum* | 84.70 |  | 5.19E-149 | AP017696 |
|  | Cg_24_3a | 545 | *Parascaris equorum* | 84.20 |  | 1.12E-145 | AP017696 |
|  | Cg_24_3b | 566 | *Parascaris equorum* | 84.50 |  | 1.43E-154 | AP017696 |
|  | Cg_8_5 | 515 | *Parascaris equorum* | 84.50 |  | 1.46E-139 | AP017696 |
| ***N. ionah*** | Ni_3_2a | 435 | *Contracaecum* cf. *osculatum* D | 98.90 |  | 0 | KF771361 |
|  | Ni_3_2b | 436 | *Contracaecum* cf. *osculatum* D | 98.90 |  | 0 | KF771361 |
|  | Ni_2_2 | 468 | *Contracaecum* cf. *osculatum* D | 98.70 |  | 0 | KT285808 |
|  | Ni_1_2.1 | 468 | *Contracaecum* cf. *osculatum* E | 99.50 |  | 0 | KF771355 |
|  | Ni_1_2 | 456 | *Contracaecum* cf. *osculatum* E | 98.70 |  | 0 | KT285809 |
|  | Ni_2_3 | 468 | *Contracaecum radiatum* | 98.70 |  | 0 | EU477210 |
| ***P. macropterus*** | Pm_3_2.1g | 421 | *Contracaecum* cf. *osculatum* D | 98.80 |  | 0 | KC412223 |
|  | Pm_4_9.2a | 460 | *Contracaecum* cf. *osculatum* D | 99.10 |  | 0 | KC412223 |
|  | Pm_1_2b | 459 | *Contracaecum* cf. *osculatum* D | 98.70 |  | 0 | KC412227 |
|  | Pm_4_2n | 446 | *Contracaecum* cf. *osculatum* D | 98.70 |  | 0 | KF771354 |
|  | Pm_3_2.1f | 445 | *Contracaecum* cf. *osculatum* D | 98.20 |  | 0 | KF771356 |
|  | Pm_1_2a | 452 | *Contracaecum* cf. *osculatum* D | 98.90 |  | 0 | KF771361 |
|  | Pm_2_2 | 458 | *Contracaecum* cf. *osculatum* D | 98.70 |  | 0 | KF771361 |
|  | Pm_3_2.1d | 441 | *Contracaecum* cf. *osculatum* D | 99.50 |  | 0 | KF771361 |
|  | Pm_4_2a | 448 | *Contracaecum* cf. *osculatum* D | 98.90 |  | 0 | KF771361 |
|  | Pm_4_2e | 448 | *Contracaecum* cf. *osculatum* D | 99.30 |  | 0 | KF771361 |
|  | Pm_4_2c | 463 | *Contracaecum* cf. *osculatum* D | 99.40 |  | 0 | KT285804 |
|  | Pm_4_2b | 459 | *Contracaecum* cf. *osculatum* E | 98.90 |  | 0 | EU477205 |
|  | Pm_3_2.1b | 465 | *Contracaecum* cf. *osculatum* E | 99.60 |  | 0 | KC412229 |
|  | Pm_4_2j | 457 | *Contracaecum* cf. *osculatum* E | 98.70 |  | 0 | KF771355 |
|  | Pm_4_2k | 457 | *Contracaecum* cf. *osculatum* E | 98.70 |  | 0 | KF771355 |
|  | Pm_1_2c | 469 | *Contracaecum* cf. *osculatum* E | 99.40 |  | 0 | KT285805 |
|  | Pm_3_2.1e | 465 | *Contracaecum* cf. *osculatum* E | 99.10 |  | 0 | KT285805 |
|  | Pm_3_9 | 459 | *Contracaecum* cf. *osculatum* E | 99.10 |  | 0 | KT285805 |
|  | Pm_4_2d | 458 | *Contracaecum* cf. *osculatum* E | 97.40 |  | 0 | KT285805 |
|  | Pm_4_2g | 433 | *Contracaecum* cf. *osculatum* E | 99.10 |  | 0 | KT285805 |
|  | Pm_4_2h | 467 | *Contracaecum* cf. *osculatum* E | 98.90 |  | 0 | KT285805 |
|  | Pm_4_2i | 457 | *Contracaecum* cf. *osculatum* E | 98.70 |  | 0 | KT285805 |
|  | Pm_4_2m | 457 | *Contracaecum* cf. *osculatum* E | 98.90 |  | 0 | KT285805 |
|  | Pm_3_2.1c | 433 | *Contracaecum* cf. *osculatum* E | 99.10 |  | 0 | KT285809 |
|  | Pm_4_2l | 438 | *Contracaecum* cf. *osculatum* E | 98.90 |  | 0 | KT285809 |
|  | Pm_2_4 | 454 | *Contracaecum osculatum* | 98.90 |  | 0 | KF718939 |
|  | Pm_4_1 | 467 | *Contracaecum osculatum* | 98.70 |  | 0 | KF718940 |
|  | Pm_4_9.2b | 419 | *Contracaecum radiatum* | 97.90 |  | 0 | EU477210 |
|  | Pm_3_2.1a | 532 | *Contracaecum* sp. | 94.20 |  | 0 | KF718925 |
| ***P. georgianus*** | Pg_15_2.7 | 471 | *Contracaecum* cf. *osculatum* D | 99.40 |  | 0 | KC412223 |
|  | Pg_1_2.1b | 473 | *Contracaecum* cf. *osculatum* D | 99.20 |  | 0 | KF771354 |
|  | Pg_12_2.2 | 468 | *Contracaecum* cf. *osculatum* D | 98.50 |  | 0 | KF771356 |
|  | Pg_8_2 | 468 | *Contracaecum* cf. *osculatum* D | 98.10 |  | 0 | KF771356 |
|  | Pg_10_2.6a | 471 | *Contracaecum* cf. *osculatum* D | 98.90 |  | 0 | KF771361 |
|  | Pg_10_9.1 | 460 | *Contracaecum* cf. *osculatum* D | 98.60 |  | 0 | KF771361 |
|  | Pg_11_4.2c | 456 | *Contracaecum* cf. *osculatum* D | 98.90 |  | 0 | KF771361 |
|  | Pg_1_2.1a | 458 | *Contracaecum* cf. *osculatum* E | 98.70 |  | 0 | KT285805 |
|  | Pg_2_4.1 | 464 | *Contracaecum* cf. *osculatum* E | 99.40 |  | 0 | KT285805 |
|  | Pg_3_2.1 | 460 | *Contracaecum* cf. *osculatum* E | 99.60 |  | 0 | KT285809 |
|  | Pg_15_2.8 | 464 | *Contracaecum osculatum* | 98.90 |  | 0 | KF718939 |
|  | Pg_5_2.1 | 471 | *Contracaecum osculatum* | 98.70 |  | 0 | KF718939 |
|  | Pg_7_2 | 469 | *Contracaecum osculatum* | 98.50 |  | 0 | KF718939 |
|  | Pg_10_2.3b | 466 | *Contracaecum radiatum* | 97.90 |  | 0 | EU477210 |
|  | Pg_11_1.1 | 452 | *Contracaecum radiatum* | 98.70 |  | 0 | EU477210 |
|  | Pg_2_3.1 | 466 | *Contracaecum radiatum* | 97.60 |  | 0 | EU477210 |
|  | Pg_9_2.2a | 458 | *Contracaecum radiatum* | 97.70 |  | 0 | EU477210 |
|  | Pg_10_5 | 534 | *Contracaecum* sp. | 93.50 |  | 0 | KF718907 |
|  | Pg_8_3.1 | 537 | *Contracaecum* sp*.* | 98.90 |  | 0 | KF718911 |
|  | Pg_9_3 | 554 | *Contracaecum* sp. | 93.00 |  | 0 | KF718911 |
|  | Pg_3_5.1 | 542 | *Contracaecum* sp. | 93.20 |  | 0 | KF718912 |
|  | Pg_6_3.2 | 534 | *Contracaecum* sp. | 92.70 |  | 0 | KF718920 |
|  | Pg_11_1 | 488 | *Contracaecum* sp. | 92.60 |  | 0 | KF718928 |
|  | Pg_11_4b | 470 | *Pseudoterranova* sp. | 97.30 |  | 0 | KF718941 |
