# Supplementary material for: Lighten up the dark: metazoan parasites as indicators for the ecology of Antarctic crocodile icefish (Channichthyidae) from the north-west Antarctic Peninsula
Source: PeerJ. 2018 May 11;6:e4638. doi: 10.7717/peerj.4638 (PMC5951144; doi:10.7717/peerj.4638)
Supplement: Supplemental Information 5 — ID = identification code of the examined fish specimens (C.w: Chaenodraco wilsoni; C.g: Champsocephalus gunnari; N.i: Neopagetopsis ionah; P.m: Pagetopsis macropterus; P.g: Pseudochaenichthys georgianus). Hol = sampling point, SL = standard length, TL = total length, TW = total weight, CW = carcass weight, GO = gonad weight, LW = liver weight, SW = stomach weight, FW = food item weight. [file peerj-06-4638-s005.docx]

**Supplemental raw data S5**: **Description of raw data including sampling information, morphological measurements and stomach contents.** ID = identification code of the examined fish specimens (C.w: Chaenodraco wilsoni; C.g: Champsocephalus gunnari; N.i: Neopagetopsis ionah; P.m: Pagetopsis macropterus; P.g: Pseudochaenichthys georgianus). Hol = sampling point, SL = standard length, TL = total length, TW = total weight, CW = carcass weight, GO = gonad weight, LW = liver weight, SW = stomach weight, FW = food item weight.

| **ID** | **Hol.** | **Catch date** | **SL (cm)** | **TL (cm)** | **TW (g)** | **CW (g)** | **GO (g)** | **LW (g)** | **SW (g)** | **SW empty (g)** | **Food items** | **FW (g)** |
| --- | --- | --- | --- | --- | --- | --- | --- | --- | --- | --- | --- | --- |
| Cw_1 | 268 | 01.04.2012 | 24.1 | 27.1 | 131.44 | 104.04 | 0.68 | 2.63 | 4.9 | 1.53 | 8 Crustacea indet. | 2.81 |
| Cw_2 | 268 | 01.04.2012 | 26.7 | 31 | 185.02 | 143.28 | 2.27 | 2.82 | 2.7 | 1.76 | no stomach content | 0.75 |
| Cw_3 | 268 | 01.04.2012 | 29.5 | 33.5 | 315.59 | 233.14 | 6.3 | 9.91 | 4.7 | 3.31 | 4 Crustacea indet. | 1.49 |
| Cw_4 | 268 | 01.04.2012 | 19.5 | 21.8 | 60.32 | 42.98 | x | 0.47 | 1.58 | 0.86 | 3 Crustacea indet. | 0.51 |
| Cw_5 | 268 | 01.04.2012 | 24.3 | 27.6 | 271 | 110.06 | 0.3 | 2.73 | 12.56 | 2.56 | 5 Crustacea indet. | 2.92 |
|  |  |  |  |  |  |  |  |  |  |  | 1 *Euphausia superba* | 8.38 |
| Cw_6 | 204 | 20.03.2012 | 20.4 | 23.5 | 85.12 | 64.07 | 0.33 | 1.76 | 2.32 | 1.2 | 4 Crustacea indet. | 1.56 |
| Cw_7 | 268 | 01.04.2012 | 28.5 | 32.4 | 305.11 | 210.18 | 7.19 | 13.84 | 7.94 | 3.03 | 10 Crustacea indet. | 4.28 |
| Cw_8 | 268 | 01.04.2012 | 28.8 | 31.7 | 249.64 | 184.53 | 0.82 | 5.34 | 3.4 | 2.55 | no stomach content | 0.79 |
| Cw_9 | 268 | 01.04.2012 | 25.3 | 28.85 | 186.52 | 138.77 | 0.33 | 4.27 | 4.44 | 2.16 | 8 *Euphausia* sp. | 1.81 |
| Cw_10 | 268 | 01.04.2012 | 29.1 | 33 | 282.92 | 227.55 | 4.54 | 7.95 | 9.36 | 4 | 17 *Euphausia* sp. | 4.34 |
| Cw_11 | 268 | 01.04.2012 | 28.2 | 32.1 | 276.73 | 197.9 | 4.32 | 6.9 | 19.74 | 3.57 | 43 *Euphausia superba* (Juvenile) | 15.87 |
| Cw_12 | 268 | 01.04.2012 | 30.05 | 34.1 | 311.69 | 238.43 | 6.06 | 9.5 | 4.53 | 3.32 | no stomach content | 0.68 |
| Cw_13 | 268 | 01.04.2012 | 24.6 | 27.9 | 161.54 | 121.14 | 2.46 | 7.5 | 5.08 | 2.02 | 7 Crustacea indet. | 2.1 |
| Cw_14 | 268 | 01.04.2012 | 31.3 | 35.1 | 346.9 | 271.06 | 8.21 | 13.16 | 4.73 | 3.42 | no stomach content | 0.1 |
| Cw_15 | 268 | 01.04.2012 | 26.7 | 30.2 | 227.7 | 178.55 | 0.82 | 7.29 | 2.99 | 2.4 | no stomach content | 0.18 |
| Cw_16 | 268 | 01.04.2012 | 29.4 | 33.3 | 268.49 | 218.65 | 4.37 | 6.55 | 3.43 | 2.59 | 3 Crustacea indet. | 0.53 |
| Cw_17 | 268 | 01.04.2012 | 28.7 | 32.4 | 259.09 | 226.28 | 5.35 | 11.03 | 3.74 | 2.86 | no stomach content | 0.56 |
| Cw_18 | 268 | 01.04.2012 | 28.3 | 32.1 | 214.33 | 176.76 | 4.39 | 2.88 | 2.63 | 1.94 | no stomach content | 0.28 |
| Cw_19 | 221 | 23.03.2012 | 20.4 | 22.5 | 81.3 | 57.78 | x | 1.76 | 8.86 | 1.16 | 27 *Euphausia* | 7.11 |
| Cw_20 | 268 | 01.04.2012 | 28.2 | 32.2 | 227.87 | 186.25 | 3.33 | 3.58 | 2.97 | 2.22 | no stomach content |  |
| Cw_21 | 268 | 01.04.2012 | 29.2 | 32.9 | 281.38 | 216.6 | 8.29 | 10.59 | 7.87 | 3.52 | 8 *Euphausia* sp. | 2.3 |
|  |  |  |  |  |  |  |  |  |  |  | 14 *Euphausia superba* | 1.7 |
| Cw_22 | 268 | 01.04.2012 | 27.8 | 31.6 | 210.22 | 169.74 | 2.95 | 2.86 | 3.11 | 2.31 | no stomach content | 0.51 |
| Cw_23 | 268 | 01.04.2012 | 29.2 | 32.7 | 289.94 | 229.09 | 5.27 | 7.64 | 6.67 | 3.15 | 14 *Euphausia* sp | 2.6 |
| Cw_24 | 268 | 01.04.2012 | 29.1 | 32.6 | 326.76 | 247.31 | 5.84 | 14.14 | 4.86 | 3.99 | no stomach content | 0.42 |
| Cw_25 | 268 | 01.04.2012 | 26.3 | 28.6 | 216.01 | 162.03 | 4.56 | 7.71 | 6.75 | 2.03 | 9 *Euphausia* sp. | 4.01 |
| Cw_26 | 268 | 01.04.2012 | 29 | 32.3 | 285.27 | 221.36 | 0.89 | 9.72 | 10.47 | 2.88 | 19 *Euphausia* sp. | 6.74 |
| Cw_27 | 268 | 01.04.2012 | 30.4 | 33.2 | 312.77 | 238.17 | 6.12 | 10.33 | 8.32 | 3.96 | 8 *Euphausia* sp. | 2.28 |
|  |  |  |  |  |  |  |  |  |  |  | 5 *Euphausia superba* | 1.77 |
| Cw_28 | 268 | 01.04.2012 | 28.6 | 32.4 | 278.84 | 215.27 | 5.92 | 8.74 | 4.02 | 2.52 | 10 Crustacea indet. | 4.9 |
| Cw_29 | 268 | 01.04.2012 | 27.2 | 30 | 224.47 | 182.67 | 0.76 | 3.57 | 4.53 | 2.01 | 7 Crustacea indet. | 2.04 |
| Cw_30 | 268 | 01.04.2012 | 28.6 | 32.2 | 257.38 | 197.5 | 6.35 | 8.24 | 5.1 | 2.3 | 5 Crustacea indet. | 1.72 |
| Cw_31 | 268 | 01.04.2012 | 26.2 | 29.1 | 190.36 | 140.34 | 0.51 | 5.87 | 5.7 | 1.54 | 10 Crustacea indet. | 2.68 |
| Cw_32 | 247 | 28.03.2012 | 19.8 | 22.3 | 66.22 | 52.09 | 0.33 | 1.03 | 4.16 | 0.8 | 4 Crustacea indet. | 2.71 |
| Cw_33 | 247 | 28.03.2012 | 13.3 | 15.4 | 16.14 | 11.2 | x | 0.18 | 1.7 | 0.2 | 4 Crustacea indet. | 1.33 |
| Cg_1 | 221 | 23.03.2012 | 30.6 | 34.5 | 273.52 | 222.9 | 0.92 | 4.9 | 5.84 | 2.4 | 10 *Euphausia* sp. | 2.96 |
| Cg_2 | 221 | 23.03.2012 | 33.4 | 37.8 | 351.78 | 239.94 | 0.91 | 5.6 | 5.55 | 3.07 | 3 Crustacea indet. | 2.1 |
| Cg_3 | 221 | 23.03.2012 | 21.4 | 24.5 | 76.26 | 64.52 | 0.5 | 0.83 | 1.3 | 0.63 | 3 Crustacea indet. | 0.65 |
| Cg_4 | 221 | 23.03.2012 | 30.7 | 34.2 | 318.48 | 247.99 | 0.75 | 5.56 | 7.11 | 2.24 | 2 *Euphausia superba* | 2.21 |
|  |  |  |  |  |  |  |  |  |  |  | 3 Crustacea indet. | 1.22 |
| Cg_5 | 221 | 23.03.2012 | 21.2 | 23.1 | 68.83 | 57.38 | 0.35 | 0.75 | 1.82 | 0.88 | 12 *Euphausia* sp. | 0.92 |
| Cg_6 | 221 | 23.03.2012 | 33.3 | 37.2 | 338.26 | 269.94 | 1.25 | 6.86 | 5.23 | 3.39 | 4 Crustacea indet. | 1.41 |
| Cg_7 | 221 | 23.03.2012 | 12.1 | 13.5 | 12.78 | 9.58 | 0.01 | 0.19 | 1.04 | 0.18 | 2 *Euphausia superba* | 0.75 |
| Cg_8 | 221 | 23.03.2012 | 23.4 | 26.4 | 101.03 | 83.4 | 0.02 | 1.34 | 3.14 | 1.13 | 24 *Euphausia* sp. | 1.69 |
| Cg_9 | 221 | 23.03.2012 | 12 | 13.2 | 12.93 | 10.4 | x | 0.1 | 0.97 | 0.11 | 2 *Euphausia superba* | 0.7 |
| Cg_10 | 221 | 23.03.2012 | 27.2 | 24.5 | 97.3 | 76.55 | 0.54 | 1.33 | 6.23 | 1.1 | 11 *Euphausia superba* | 3.08 |
|  |  |  |  |  |  |  |  |  |  |  | 36 *Euphausia* sp. | 1.46 |
| Cg_11 | 221 | 23.03.2012 | 31.5 | 35 | 308.38 | 254.05 | 0.64 | 5.97 | 3.76 | 3.45 | no stomach content | 0.47 |
| Cg_12 | 221 | 23.03.2012 | 25.2 | 28.7 | 112.68 | 89.07 | 0.08 | 1.21 | 3.3 | 1.06 | 7 *Euphausia* sp. | 1.91 |
|  |  |  |  |  |  |  |  |  |  |  | 1 Amphipoda | 0.04 |
| Cg_13 | 221 | 23.03.2012 | 20.5 | 23.3 | 71.74 | 57.82 | 0.28 | 0.86 | 3.9 | 0.84 | 34 *Euphausia* sp. | 2.82 |
| Cg_14 | 221 | 23.03.2012 | 30.2 | 34.1 | 186.4 | 149.1 | 1.88 | 5.42 | 8.58 | 2.32 | 17 *Euphausia* sp. | 5.86 |
| Cg_15 | 221 | 23.03.2012 | 31.7 | 36.2 | 314.14 | 252.79 | 0.87 | 5.58 | 4.57 | 2.92 | 1 Crustacea indet. | 1.19 |
| Cg_16 | 221 | 23.03.2012 | 31.6 | 35.9 | 306.56 | 237.24 | 0.68 | 6.67 | 9.01 | 3.03 | 14 Crustacea indet. | 3.98 |
|  |  |  |  |  |  |  |  |  |  |  | 1 *Euphausia superba* | 0.88 |
| Cg_17 | 221 | 23.03.2012 | 33.8 | 37.3 | 369.35 | 280.08 | 0.43 | 5.97 | 29.25 | 4.48 | 43 *Euphausia superba* | 20.28 |
|  |  |  |  |  |  |  |  |  |  |  | 8 *Euphausia* sp. | 1.8 |
| Cg_18 | 221 | 23.03.2012 | 21.3 | 24.4 | 89.28 | 70.12 | 0.45 | 2.3 | 3.61 | 0.87 | 5 *Euphausia* sp | 1.62 |
|  |  |  |  |  |  |  |  |  |  |  | 6 Crustacea indet. | 0.87 |
| Cg_19 | 221 | 23.03.2012 | 25.4 | 28.7 | 135.17 | 104.87 | 0.75 | 1.88 | 8.75 | 1.42 | 14 *Euphausia* sp. | 6.2 |
| Cg_20 | 221 | 23.03.2012 | 26.2 | 29.5 | 141.62 | 108.57 | 1.72 | 2.83 | 5.74 | 1.58 | 3 *Euphausia* sp. | 2.98 |
|  |  |  |  |  |  |  |  |  |  |  | 7 Crustacea indet. | 0.87 |
| Cg_21 | 221 | 23.03.2012 | 24.9 | 28.1 | 135.71 | 107.75 | 0.18 | 2 | 6.91 | 1.67 | 13 *Euphausia* sp. | 4.24 |
| Gg_22 | 221 | 23.03.2012 | 31 | 34.5 | 235.6 | 203.71 | 2.53 | 6.08 | 16.4 | 3.32 | 33 *Euphausia* sp. | 11.49 |
|  |  |  |  |  |  |  |  |  |  |  | 5 Amphipoda | 0.31 |
| Cg_23 | 221 | 23.03.2012 | 32.5 | 36.5 | 411.98 | 318.97 | 28.4 | 15.72 | 17.2 | 3.79 | 32 *Euphausia* sp. | 11.24 |
| Cg_24 | 221 | 23.03.2012 | 28.2 | 31.5 | 169.7 | 122.4 | 1.48 | 3.26 | 6.01 | 1.78 | 19 *Euphausia* sp. | 3.37 |
| Cg_25 | 221 | 23.03.2012 | 29.5 | 33.1 | 267.73 | 203.99 | 0.38 | 3.84 | 18.88 | 3.053 | 34 *Euphausia superba* | 13.24 |
|  |  |  |  |  |  |  |  |  |  |  |  |  |
| Ni_1 | 265 | 31.03.2012 | 52.5 | 54.7 | 1237.54 | 951.13 | 12.33 | 38.99 | 86.53 | 18.977 | 53 *Euphausia* sp. | 47.56 |
| Ni_2 | 242 | 27.03.2012 | 44.5 | 50.8 | 1060.89 | 830.32 | 15.98 | 32.14 | 25.22 | 10.39 | 16 Crustacea indet. | 4.29 |
| Ni_3 | 242 | 27.03.2012 | 42.4 | 51.4 | 1142.5 | 839.3 | 10.33 | 27.31 | 17.99 | 14.02 | no stomach content | 1.15 |
|  |  |  |  |  |  |  |  |  |  |  |  |  |
| Pm_1 | 269 | 01.04.2012 | 17.3 | 18.6 | 57.38 | 43.4 | 0.12 | 1.22 | 2.75 | 0.87 | 2 Crustacea indet. | 0.86 |
| Pm_2 | 269 | 01.04.2012 | 22.4 | 25.4 | 149.26 | 109.3 | 0.79 | 3.85 | 11 | 2.83 | 14 *Euphausia* sp. | 6.56 |
| Pm_3 | 269 | 01.04.2012 | 20.7 | 23.5 | 91.44 | 69.24 | 0.39 | 1.95 | 4.49 | 1.93 | 2 Crustacea indet. | 2.12 |
| Pm_4 | 269 | 01.04.2012 | 20.8 | 23.5 | 90.29 | 62.47 | 0.17 | 2.06 | 10.73 | 2.32 | 19 Crustacea indet. | 6.15 |
|  |  |  |  |  |  |  |  |  |  |  |  |  |
| Pg_1 | 253 | 29.03.2012 | 29.5 | 34.2 | 316.35 | 239.28 | 1 | 6.89 | 13.34 | 8.57 | no stomach content | x |
| Pg_2 | 253 | 29.03.2012 | 30.9 | 35.1 | 368.12 | 257.45 | 1.12 | 9.81 | 10.07 | 8.22 | no stomach content | x |
| Pg_3 | 209 | 24.03.2012 | 32.3 | 36.6 | 428.9 | 318.85 | 1.15 | 12.85 | 14.8 | 10.78 | no stomach content | x |
| Pg_4 | 209 | 24.03.2012 | 32.5 | 37.2 | 389.4 | 306.36 | 1.49 | 10.49 | 14.96 | 12.36 | no stomach content | x |
| Pg_5 | 259 | 30.03.2012 | 28.3 | 33.2 | 227.32 | 236.49 | 1.16 | 8.4 | 9.93 | 7.61 | no stomach content | x |
| Pg_6 | 259 | 30.03.2012 | 39.6 | 45.1 | 886.39 | 663.18 | 5.58 | 36.47 | 28.63 | 24.19 | no stomach content | x |
| Pg_7 | 259 | 30.03.2012 | 19.2 | 22.3 | 65.04 | 48.24 | x | 1.29 | 1.89 | 1.25 | 1 Crustacea indet. | 0.28 |
| Pg_8 | 257 | 30.03.2012 | 29.9 | 34.9 | 369.76 | 275.11 | 0.01 | 10.37 | 12.04 | 8.49 | no stomach content | x |
| Pg_9 | 257 | 30.03.2012 | 39.4 | 44.3 | 1097.28 | 647.31 | 5.48 | 34.03 | 31.63 | 28.08 | 2 *Euphausia* sp. | 1.08 |
| Pg_10 | 265 | 31.03.2012 | 41.1 | 46.3 | 1199.25 | 882.13 | 16.26 | 48.4 | 34.5 | 32.42 | no stomach content | x |
| Pg_11 | 209 | 21.02.2012 | 42.4 | 47.2 | 1639.61 | 837.53 | 6.1 | 39.98 | 46.3 | 45.16 | no stomach content | x |
| Pg_12 | 220 | 23.03.2012 | 30.9 | 33.6 | 406.41 | 265.96 | 0.15 | 7.59 | 14.35 | 8.82 | no stomach content | x |
| Pg_13 | 204 | 20.03.2012 | 30.2 | 35.1 | 465.65 | 340.8 | 1.06 | 13.2 | 19.94 | 14.06 | 2 Crustacea indet. | 1.97 |
| Pg_14 | 204 | 20.03.2012 | 20.9 | 24.2 | 111.05 | 79.39 | 0.14 | 1.51 | 15.36 | 2.3 | 21 *Euphausia* sp. | 10.65 |
| Pg_15 | 266 | 31.03.2012 | 38.7 | 40.5 | 741.8 | 551.91 | 3.84 | 27.6 | 0 | 24 | no stomach content | x |
