# Supplementary material for: Lighten up the dark: metazoan parasites as indicators for the ecology of Antarctic crocodile icefish (Channichthyidae) from the north-west Antarctic Peninsula
Source: PeerJ. 2018 May 11;6:e4638. doi: 10.7717/peerj.4638 (PMC5951144; doi:10.7717/peerj.4638)
Supplement: Supplemental Information 6 — Given numbers are the amount of recorded parasite specimens. ID = identification code of the examined specimens. Hir: Hirudinea, N. saw: Nototheniobdella sawyeri, N. noto: Notobdella nototheniae, T. cap: Truliobdella capitis, Ne: Nematoda, C. osc: Contracaecum osculatum (s.l.), C. rad: C. radiatum, C. sp.: Contracaecum sp., P. dec: Pseudoterranova decipiens (s.l.), A. not: Ascarophis nototheniae, Dig: Digenea, N. ant: Neolebouria antarctica, M. geo: Macvicaria georgiana, G. phy: Gonocerca phycidis, E. oat: Elytrophalloides oatesi, Ces: Cestoda, Db: Diphyllobothriidea, Te: Tetraphyllidae, Ac: Acanthocephala, C. bul: Corynosoma bullosum, Cr: Crustacea, E. ant: Eubrachiella antarctica. [file peerj-06-4638-s006.docx]

**Supplemental Raw Data S6: Raw data of parasitological examination.** Given numbers are the amount of recorded parasite specimen. ID = identification code of the examined specimens. Hir: Hirudinea, N. saw: Nototheniobdella sawyeri, N. noto: Notobdella nototheniae, T. cap: Trulliobdella capitis, Ne: Nematoda, C. osc: Contracaecum osculatum (s.l.), C. rad: C. radiatum, C. sp.: Contracaecum sp., P. dec: Pseudoterranova decipiens (s.l.), A. not: Ascarophis nototheniae, Dig: Digenea, N. ant: Neolebouria antarctica, M. geo: Macvicaria georgiana, G. phy: Gonocerca phycidis, E. oat: Elytrophalloides oatesi, Ces: Cestoda, Ps: Pseudophyllidae, Te: Tetraphyllidae, Ac: Acanthocephala, C. bul: Corynosoma bullosum, Cr: Crustacea, E. ant: Eubrachiella antarctica.

| **ID** | **Hir** | ***N. saw*** | ***N. not*** | ***T. cap*** | ***Ne.*** | ***C. osc*** | ***C. rad*** | ***C.* sp.** | ***P. dec*** | ***A. not*** | **Ne. indet.** | **Dig** | ***N ant*** | ***M. geo*** | ***G. phy*** | ***E oat*** | ***Ces*** | **Ps indet.** | **Te indet.** | ***Ac*** | ***C. bul*** | ***Cr*** | ***E. ant*** |
| --- | --- | --- | --- | --- | --- | --- | --- | --- | --- | --- | --- | --- | --- | --- | --- | --- | --- | --- | --- | --- | --- | --- | --- |
| C.w1 | **0** | 0 | 0 | 0 | **0** | 0 | 0 | 0 | 0 | 0 | 0 | **0** | 0 | 0 | 0 | 0 | **0** | 0 | 0 | **0** | 0 | **0** | 0 |
| C.w2 | **0** | 0 | 0 | 0 | **0** | 0 | 0 | 0 | 0 | 0 | 0 | **0** | 0 | 0 | 0 | 0 | **0** | 0 | 0 | **0** | 0 | **0** | 0 |
| C.w3 | **2** | 2 | 0 | 0 | **0** | 0 | 0 | 0 | 0 | 0 | 0 | **0** | 0 | 0 | 0 | 0 | **0** | 0 | 0 | **0** | 0 | **0** | 0 |
| C.w4 | **0** | 0 | 0 | 0 | **1** | 0 | 1 | 0 | 0 | 0 | 0 | **0** | 0 | 0 | 0 | 0 | **0** | 0 | 0 | **0** | 0 | **0** | 0 |
| C.w5 | **0** | 0 | 0 | 0 | **1** | 0 | 1 | 0 | 0 | 0 | 0 | **0** | 0 | 0 | 0 | 0 | **0** | 0 | 0 | **0** | 0 | **0** | 0 |
| C.w6 | **0** | 0 | 0 | 0 | **0** | 0 | 0 | 0 | 0 | 0 | 0 | **0** | 0 | 0 | 0 | 0 | **0** | 0 | 0 | **0** | 0 | **0** | 0 |
| C.w7 | **0** | 0 | 0 | 0 | **1** | 1 | 0 | 0 | 0 | 0 | 0 | **0** | 0 | 0 | 0 | 0 | **0** | 0 | 0 | **0** | 0 | **0** | 0 |
| C.w8 | **21** | 1 | 20 | 0 | **0** | 0 | 0 | 0 | 0 | 0 | 0 | **2** | 2 | 0 | 0 | 0 | **0** | 0 | 0 | **0** | 0 | **0** | 0 |
| C.w9 | **0** | 0 | 0 | 0 | **0** | 0 | 0 | 0 | 0 | 0 | 0 | **0** | 0 | 0 | 0 | 0 | **0** | 0 | 0 | **0** | 0 | **0** | 0 |
| C.w10 | **0** | 0 | 0 | 0 | **0** | 0 | 0 | 0 | 0 | 0 | 0 | **0** | 0 | 0 | 0 | 0 | **0** | 0 | 0 | **0** | 0 | **0** | 0 |
| C.w11 | **0** | 0 | 0 | 0 | **0** | 0 | 0 | 0 | 0 | 0 | 0 | **0** | 0 | 0 | 0 | 0 | **0** | 0 | 0 | **0** | 0 | **0** | 0 |
| C.w12 | **1** | 0 | 0 | 1 | **4** | 1 | 3 | 0 | 0 | 0 | 0 | **1** | 1 | 0 | 0 | 0 | **0** | 0 | 0 | **0** | 0 | **0** | 0 |
| C.w13 | **0** | 0 | 0 | 0 | **0** | 0 | 0 | 0 | 0 | 0 | 0 | **0** | 0 | 0 | 0 | 0 | **0** | 0 | 0 | **0** | 0 | **0** | 0 |
| C.w14 | **0** | 0 | 0 | 0 | **11** | 8 | 1 | 0 | 0 | 0 | 2 | **0** | 0 | 0 | 0 | 0 | **0** | 0 | 0 | **0** | 0 | **0** | 0 |
| C.w15 | **6** | 1 | 5 | 0 | **0** | 0 | 0 | 0 | 0 | 0 | 0 | **0** | 0 | 0 | 0 | 0 | **0** | 0 | 0 | **0** | 0 | **0** | 0 |
| C.w16 | **0** | 0 | 0 | 0 | **0** | 0 | 0 | 0 | 0 | 0 | 0 | **0** | 0 | 0 | 0 | 0 | **0** | 0 | 0 | **0** | 0 | **0** | 0 |
| C.w17 | **0** | 0 | 0 | 0 | **1** | 1 | 0 | 0 | 0 | 0 | 0 | **0** | 0 | 0 | 0 | 0 | **0** | 0 | 0 | **0** | 0 | **0** | 0 |
| C.w18 | **2** | 0 | 2 | 0 | **2** | 2 | 0 | 0 | 0 | 0 | 0 | **0** | 0 | 0 | 0 | 0 | **0** | 0 | 0 | **0** | 0 | **0** | 0 |
| C.w19 | **0** | 0 | 0 | 0 | **5** | 4 | 1 | 0 | 0 | 0 | 0 | **0** | 0 | 0 | 0 | 0 | **0** | 0 | 0 | **0** | 0 | **0** | 0 |
| C.w20 | **0** | 0 | 0 | 0 | **5** | 2 | 2 | 0 | 0 | 0 | 1 | **0** | 0 | 0 | 0 | 0 | **0** | 0 | 0 | **0** | 0 | **0** | 0 |
| C.w21 | **2** | 2 | 0 | 0 | **2** | 2 | 0 | 0 | 0 | 0 | 0 | **0** | 0 | 0 | 0 | 0 | **0** | 0 | 0 | **0** | 0 | **0** | 0 |
| C.w22 | **2** | 1 | 0 | 1 | **16** | 7 | 2 | 6 | 0 | 0 | 1 | **0** | 0 | 0 | 0 | 0 | **2** | 2 | 0 | **0** | 0 | **0** | 0 |
| C.w23 | **0** | 0 | 0 | 0 | **4** | 3 | 0 | 0 | 0 | 0 | 1 | **0** | 0 | 0 | 0 | 0 | **0** | 0 | 0 | **0** | 0 | **0** | 0 |
| C.w24 | **0** | 0 | 0 | 0 | **3** | 2 | 1 | 0 | 0 | 0 | 0 | **0** | 0 | 0 | 0 | 0 | **3** | 3 | 0 | **0** | 0 | **0** | 0 |
| C.w25 | **0** | 0 | 0 | 0 | **0** | 0 | 0 | 0 | 0 | 0 | 0 | **0** | 0 | 0 | 0 | 0 | **0** | 0 | 0 | **0** | 0 | **0** | 0 |
| C.w26 | **5** | 1 | 4 | 0 | **0** | 0 | 0 | 0 | 0 | 0 | 0 | **0** | 0 | 0 | 0 | 0 | **0** | 0 | 0 | **0** | 0 | **0** | 0 |
| C.w27 | **0** | 0 | 0 | 0 | **0** | 0 | 0 | 0 | 0 | 0 | 0 | **0** | 0 | 0 | 0 | 0 | **0** | 0 | 0 | **0** | 0 | **0** | 0 |
| C.w28 | **1** | 0 | 1 | 0 | **0** | 0 | 0 | 0 | 0 | 0 | 0 | **0** | 0 | 0 | 0 | 0 | **0** | 0 | 0 | **0** | 0 | **0** | 0 |
| C.w29 | **0** | 0 | 0 | 0 | **1** | 0 | 1 | 0 | 0 | 0 | 0 | **0** | 0 | 0 | 0 | 0 | **1** | 1 | 0 | **0** | 0 | **0** | 0 |
| C.w30 | **1** | 1 | 0 | 0 | **0** | 0 | 0 | 0 | 0 | 0 | 0 | **0** | 0 | 0 | 0 | 0 | **0** | 0 | 0 | **0** | 0 | **0** | 0 |
| C.w31 | **0** | 0 | 0 | 0 | **4** | 1 | 3 | 0 | 0 | 0 | 0 | **0** | 0 | 0 | 0 | 0 | **4** | 4 | 0 | **0** | 0 | **0** | 0 |
| C.w32 | **0** | 0 | 0 | 0 | **2** | 1 | 1 | 0 | 0 | 0 | 0 | **0** | 0 | 0 | 0 | 0 | **0** | 0 | 0 | **0** | 0 | **0** | 0 |
| C.w33 | **0** | 0 | 0 | 0 | **0** | 0 | 0 | 0 | 0 | 0 | 0 | **0** | 0 | 0 | 0 | 0 | **0** | 0 | 0 | **0** | 0 | **0** | 0 |
|  |  |  |  |  |  |  |  |  |  |  |  |  |  |  |  |  |  |  |  |  |  |  |  |
|  |  |  |  |  |  |  |  |  |  |  |  |  |  |  |  |  |  |  |  |  |  |  |  |
| C.g1 | **0** | 0 | 0 | 0 | **1** | 0 | 0 | 0 | 0 | 0 | 1 | **0** | 0 | 0 | 0 | 0 | **1** | 1 | 0 | **0** | 0 | **0** | 0 |
| C.g2 | **0** | 0 | 0 | 0 | **5** | 2 | 2 | 0 | 0 | 0 | 1 | **0** | 0 | 0 | 0 | 0 | **0** | 0 | 0 | **0** | 0 | **0** | 0 |
| C.g3 | **0** | 0 | 0 | 0 | **0** | 0 | 0 | 0 | 0 | 0 | 0 | **0** | 0 | 0 | 0 | 0 | **0** | 0 | 0 | **0** | 0 | **0** | 0 |
| C.g4 | **0** | 0 | 0 | 0 | **2** | 2 | 0 | 0 | 0 | 0 | 0 | **0** | 0 | 0 | 0 | 0 | **2** | 2 | 0 | **0** | 0 | **0** | 0 |
| C.g5 | **0** | 0 | 0 | 0 | **0** | 0 | 0 | 0 | 0 | 0 | 0 | **0** | 0 | 0 | 0 | 0 | **0** | 0 | 0 | **0** | 0 | **0** | 0 |
| C.g6 | **0** | 0 | 0 | 0 | **0** | 0 | 0 | 0 | 0 | 0 | 0 | **0** | 0 | 0 | 0 | 0 | **0** | 0 | 0 | **0** | 0 | **0** | 0 |
| C.g7 | **0** | 0 | 0 | 0 | **0** | 0 | 0 | 0 | 0 | 0 | 0 | **0** | 0 | 0 | 0 | 0 | **0** | 0 | 0 | **0** | 0 | **0** | 0 |
| C.g8 | **0** | 0 | 0 | 0 | **1** | 0 | 0 | 0 | 0 | 0 | 1 | **0** | 0 | 0 | 0 | 0 | **1** | 1 | 0 | **0** | 0 | **0** | 0 |
| C.g9 | **0** | 0 | 0 | 0 | **0** | 0 | 0 | 0 | 0 | 0 | 0 | **0** | 0 | 0 | 0 | 0 | **0** | 0 | 0 | **0** | 0 | **0** | 0 |
| C.g10 | **0** | 0 | 0 | 0 | **2** | 1 | 0 | 0 | 0 | 0 | 1 | **0** | 0 | 0 | 0 | 0 | **0** | 0 | 0 | **0** | 0 | **0** | 0 |
| C.g11 | **0** | 0 | 0 | 0 | **3** | 1 | 1 | 0 | 0 | 0 | 1 | **0** | 0 | 0 | 0 | 0 | **0** | 0 | 0 | **0** | 0 | **0** | 0 |
| C.g12 | **0** | 0 | 0 | 0 | **4** | 2 | 0 | 1 | 0 | 0 | 1 | **0** | 0 | 0 | 0 | 0 | **0** | 0 | 0 | **0** | 0 | **0** | 0 |
| C.g13 | **0** | 0 | 0 | 0 | **1** | 0 | 1 | 0 | 0 | 0 | 0 | **0** | 0 | 0 | 0 | 0 | **0** | 0 | 0 | **0** | 0 | **0** | 0 |
| C.g14 | **0** | 0 | 0 | 0 | **0** | 0 | 0 | 0 | 0 | 0 | 0 | **0** | 0 | 0 | 0 | 0 | **0** | 0 | 0 | **0** | 0 | **0** | 0 |
| C.g15 | **0** | 0 | 0 | 0 | **1** | 0 | 0 | 0 | 0 | 0 | 1 | **0** | 0 | 0 | 0 | 0 | **0** | 0 | 0 | **0** | 0 | **0** | 0 |
| C.g16 | **0** | 0 | 0 | 0 | **2** | 1 | 1 | 0 | 0 | 0 | 0 | **0** | 0 | 0 | 0 | 0 | **0** | 0 | 0 | **0** | 0 | **0** | 0 |
| C.g17 | **0** | 0 | 0 | 0 | **3** | 2 | 1 | 0 | 0 | 0 | 0 | **1** | 0 | 1 | 0 | 0 | **0** | 0 | 0 | **0** | 0 | **0** | 0 |
| C.g18 | **0** | 0 | 0 | 0 | **2** | 1 | 0 | 0 | 0 | 0 | 1 | **1** | 0 | 1 | 0 | 0 | **2** | 2 | 0 | **0** | 0 | **0** | 0 |
| C.g19 | **0** | 0 | 0 | 0 | **2** | 0 | 0 | 2 | 0 | 0 | 0 | **0** | 0 | 0 | 0 | 0 | **0** | 0 | 0 | **0** | 0 | **0** | 0 |
| C.g20 | **0** | 0 | 0 | 0 | **7** | 1 | 0 | 0 | 0 | 0 | 6 | **0** | 0 | 0 | 0 | 0 | **0** | 0 | 0 | **0** | 0 | **0** | 0 |
| C.g21 | **0** | 0 | 0 | 0 | **1** | 0 | 0 | 0 | 0 | 0 | 1 | **0** | 0 | 0 | 0 | 0 | **0** | 0 | 0 | **0** | 0 | **0** | 0 |
| C.g22 | **0** | 0 | 0 | 0 | **1** | 0 | 1 | 0 | 0 | 0 | 0 | **0** | 0 | 0 | 0 | 0 | **0** | 0 | 0 | **0** | 0 | **0** | 0 |
| C.g23 | **0** | 0 | 0 | 0 | **6** | 0 | 0 | 1 | 0 | 0 | 5 | **0** | 0 | 0 | 0 | 0 | **2** | 2 | 0 | **0** | 0 | **0** | 0 |
| C.g24 | **0** | 0 | 0 | 0 | **2** | 0 | 0 | 0 | 0 | 0 | 2 | **0** | 0 | 0 | 0 | 0 | **0** | 0 | 0 | **0** | 0 | **0** | 0 |
| C.g25 | **0** | 0 | 0 | 0 | **5** | 1 | 3 | 1 | 0 | 0 | 0 | **0** | 0 | 0 | 0 | 0 | **1** | 1 | 0 | **0** | 0 | **0** | 0 |
|  |  |  |  |  |  |  |  |  |  |  |  |  |  |  |  |  |  |  |  |  |  |  |  |
|  |  |  |  |  |  |  |  |  |  |  |  |  |  |  |  |  |  |  |  |  |  |  |  |
| N.i1 | **0** | 0 | 0 | 0 | **3** | 3 | 0 | 0 | 0 | 0 | 0 | **0** | 0 | 0 | 0 | 0 | **3** | 3 | 0 | **0** | 0 | **0** | 0 |
| N.i2 | **0** | 0 | 0 | 0 | **4** | 2 | 1 | 1 | 0 | 0 | 0 | **0** | 0 | 0 | 0 | 0 | **2** | 2 | 0 | **0** | 0 | **0** | 0 |
| N.i3 | **0** | 0 | 0 | 0 | **2** | 1 | 1 | 0 | 0 | 0 | 0 | **0** | 0 | 0 | 0 | 0 | **0** | 0 | 0 | **0** | 0 | **0** | 0 |
|  |  |  |  |  |  |  |  |  |  |  |  |  |  |  |  |  |  |  |  |  |  |  |  |
|  |  |  |  |  |  |  |  |  |  |  |  |  |  |  |  |  |  |  |  |  |  |  |  |
| Pm1 | **0** | 0 | 0 | 0 | **4** | 3 | 1 | 0 | 0 | 0 | 0 | **0** | 0 | 0 | 0 | 0 | **9** | 9 | 0 | **0** | 0 | **0** | 0 |
| Pm2 | **1** | 1 | 0 | 0 | **4** | 3 | 1 | 0 | 0 | 0 | 0 | **0** | 0 | 0 | 0 | 0 | **34** | 34 | 0 | **0** | 0 | **0** | 0 |
| Pm3 | **0** | 0 | 0 | 0 | **11** | 9 | 2 | 0 | 0 | 0 | 0 | **0** | 0 | 0 | 0 | 0 | **7** | 7 | 0 | **0** | 0 | **0** | 0 |
| Pm4 | **3** | 3 | 0 | 0 | **25** | 19 | 6 | 0 | 0 | 0 | 0 | **0** | 0 | 0 | 0 | 0 | **15** | 15 | 0 | **0** | 0 | **0** | 0 |
|  |  |  |  |  |  |  |  |  |  |  |  |  |  |  |  |  |  |  |  |  |  |  |  |
|  |  |  |  |  |  |  |  |  |  |  |  |  |  |  |  |  |  |  |  |  |  |  |  |
| P.g1 | **0** | 0 | 0 | 0 | **21** | 17 | 4 | 0 | 0 | 0 | 0 | **0** | 0 | 0 | 0 | 0 | **13** | 7 | 6 | **0** | 0 | **0** | 0 |
| P.g2 | **0** | 0 | 0 | 0 | **52** | 35 | 17 | 0 | 0 | 0 | 0 | **1** | 0 | 0 | 1 | 0 | **25** | 6 | 19 | **0** | 0 | **0** | 0 |
| P.g3 | **0** | 0 | 0 | 0 | **16** | 8 | 8 | 0 | 0 | 0 | 0 | **0** | 0 | 0 | 0 | 0 | **61** | 25 | 36 | **0** | 0 | **0** | 0 |
| P.g4 | **0** | 0 | 0 | 0 | **14** | 5 | 8 | 1 | 0 | 0 | 0 | **0** | 0 | 0 | 0 | 0 | **112** | 20 | 92 | **0** | 0 | **0** | 0 |
| P.g5 | **1** | 1 | 0 | 0 | **21** | 7 | 14 | 0 | 0 | 0 | 0 | **0** | 0 | 0 | 0 | 0 | **148** | 0 | 148 | **0** | 0 | **0** | 0 |
| P.g6 | **3** | 3 | 0 | 0 | **55** | 31 | 18 | 2 | 2 | 1 | 1 | **0** | 0 | 0 | 0 | 0 | **15** | 11 | 4 | **0** | 0 | **0** | 0 |
| P.g7 | **0** | 0 | 0 | 0 | **3** | 3 | 0 | 0 | 0 | 0 | 0 | **0** | 0 | 0 | 0 | 0 | **51** | 0 | 51 | **0** | 0 | **0** | 0 |
| P.g8 | **0** | 0 | 0 | 0 | **18** | 11 | 7 | 0 | 0 | 0 | 0 | **0** | 0 | 0 | 0 | 0 | **131** | 0 | 131 | **0** | 0 | **0** | 0 |
| P.g9 | **1** | 0 | 0 | 1 | **58** | 24 | 18 | 0 | 16 | 0 | 0 | **0** | 0 | 0 | 0 | 0 | **111** | 16 | 95 | **1** | 1 | **0** | 0 |
| P.g10 | **2** | 1 | 0 | 1 | **84** | 54 | 21 | 4 | 5 | 0 | 0 | **2** | 1 | 0 | 0 | 1 | **395** | 157 | 238 | **0** | 0 | **1** | 1 |
| P.g11 | **0** | 0 | 0 | 0 | **60** | 22 | 34 | 1 | 3 | 0 | 0 | **0** | 0 | 0 | 0 | 0 | **275** | 170 | 105 | **3** | 3 | **0** | 0 |
| P.g12 | **0** | 0 | 0 | 0 | **11** | 8 | 2 | 1 | 0 | 0 | 0 | **0** | 0 | 0 | 0 | 0 | **56** | 17 | 39 | **0** | 0 | **0** | 0 |
| P.g13 | **0** | 0 | 0 | 0 | **3** | 2 | 1 | 0 | 0 | 0 | 0 | **0** | 0 | 0 | 0 | 0 | **107** | 19 | 88 | **0** | 0 | **0** | 0 |
| P.g14 | **0** | 0 | 0 | 0 | **0** | 0 | 0 | 0 | 0 | 0 | 0 | **0** | 0 | 0 | 0 | 0 | **20** | 1 | 19 | **0** | 0 | **0** | 0 |
| P.g15 | **1** | 1 | 0 | 0 | **44** | 13 | 19 | 4 | 7 | 0 | 1 | **4** | 0 | 0 | 0 | 4 | **254** | 163 | 91 | **1** | 1 | **0** | 0 |
